# Electro-clinical correlation of rinch and *peri*-ictal vegetative symptoms

**DOI:** 10.1016/j.ebr.2025.100831

**Published:** 2025-10-09

**Authors:** Divya Nagabushana, Francesco Pucci, Huan Huynh, Julia Bodnya, Anna Serafini

**Affiliations:** a Epilepsy Division, Department of Neurology and Rehabilitation, University of Illinois Chicago, Chicago, USA; b Department of Neurosurgery, University of Illinois Chicago, Chicago, USA

**Keywords:** Ictal spitting, Rhythmic ictal non-clonic hand motions, Temporal lobe epilepsy, Peri-ictal vegetative symptoms, Invasive EEG, Automatisms

## Abstract

Rhythmic ictal non-clonic hand (RINCH) movements and *peri*-ictal vegetative symptoms (PIVS) are rare semiological signs in temporal lobe epilepsy (TLE). RINCH refers to rhythmic, low-amplitude, complex hand movements that typically lateralize to the contralateral hemisphere, while PIVS includes manifestations such as ictal spitting and post-ictal coughing, more frequently associated with non-dominant TLE. We report a unique case of a 40-year-old woman with drug-resistant dominant TLE who exhibited both RINCH and PIVS as part of her habitual seizures. Non-invasive EEG and imaging localized the seizure onset to the left anterior temporal region. Stereoelectroencephalography (SEEG) confirmed seizure onset in the left mesial temporal pole with rapid propagation to the hippocampus, amygdala, and other limbic structures. Notably, RINCH was observed only in seizures that showed ictal spread to the superior temporal gyrus (STG) and superior temporal sulcus (STS), suggesting their role in mediating semi-automatic motor behaviors. Ictal spitting and other PIVS were linked to the spread to the entorhinal and parahippocampal regions. The patient underwent left anterior temporal lobectomy with histopathological confirmation of hippocampal sclerosis and has remained seizure-free at one-year follow-up. This case provides a rare anatomo-electro-clinical correlation of RINCH and PIVS using SEEG and emphasizes that while RINCH retains lateralizing value, PIVS may not. Our findings underscore the importance of invasive EEG in characterizing complex semiology and refining the epileptogenic zone in dominant TLE.

## Highlights

• RINCH and PIVS co-occurred in dominant temporal lobe epilepsy (TLE).
• RINCH lateralized to the hemisphere contralateral to seizure onset.
• RINCH appeared only with spread to STG and STS on SEEG.
• PIVS like spitting and coughing lacked lateralizing value.
• Invasive EEG helped refine seizure localization for surgical planning.

## 1 Introduction

Peri-ictal vegetative symptoms, such as coughing, spitting, and post-ictal nose wiping, are rare and can have certain value in lateralization or localization [[1], [2], [3]]. Peri-ictal spitting has been described in 0.3 % of epilepsy patients and 1 % of cases with temporal lobe epilepsy (TLE) [4]. It has often been associated with non-dominant TLE. Though some of the vegetative symptoms, such as post-ictal nose wiping, post-ictal cough, and throat clearing, have been described in temporal lobe epilepsy, other data show that they are not specific to a temporal lobe onset [5,6]. Rhythmic ictal non-clonic hand (RINCH) motions, first described by Lee et al., are rhythmic, low-amplitude, complex movements that lateralize to the contralateral temporal lobe [7,8]. RINCH has been reported in 2–5 % of patients with temporal lobe epilepsy undergoing invasive evaluation and 6–8 % in general epilepsy monitoring unit (EMU) cohorts [[7], [8], [9]]. However, more robust data post-intracranial recording would help to understand these phenomena better. Very few cases in the literature demonstrate the anatomo-electro-clinical correlation of these semiological signs following intracranial EEG and surgical resection of the epileptogenic zone (EZ). The confidence level in localizing the EZ is determined by MRI findings, intracranial EEG results, and the postoperative outcomes, as outlined in a recently proposed grading system [10]. We describe the clinical features, imaging, and invasive EEG findings along with postoperative histopathological findings and 1-year follow-up of a patient with drug-resistant seizures with both RINCH and *peri*-ictal vegetative symptoms.

## 2 Case Summary

A 40-year-old right-handed woman presented with a history of drug-resistant epilepsy that started at the age of 9 years. Her seizures were characterized by loss of awareness, repeated spitting, and automatisms of the left hand. No aura reported. She was having 1–2 seizures per month despite being on 3 antiseizure medications (ASMs): levetiracetam (3 gm/day), carbamazepine (800 mg/day), and clobazam (5 mg/day). Her past medical history was unremarkable.

A 4-day phase 1 EMU admission recorded 4 seizures, all arising from the left anterior temporal region. Interictal epileptiform discharges were seen in the same region. All the seizures were stereotyped and characterized by impaired awareness followed by recurrent spitting (noted after 6–14 s). RINCH of the right upper extremity was observed 14–20 s after the EEG onset, followed by stiffening and fisting with dystonic posturing of the right hand. Intermittent cough and repetitive nose/mouth wiping automatisms with the left upper extremity were also observed. During the seizure, she could follow simple commands but was unable to speak. Postictal cough and amnesia of the event was noted at the end. Ictal onset was characterized by a rhythmic 3–4 Hz activity, seen in F7, T9, and T7 electrodes, which quickly spread to the left posterior temporal and parietal region. Seizures lasted 2–3 min.

A 3-Tesla epilepsy protocol brain MRI showed a subtle asymmetry with decreased volume of the left hippocampus relative to the right. FDG PET CT of the brain revealed hypometabolism of the anterior to mid-left lateral temporal region. Neuropsychological evaluation documented a mild neurocognitive disorder. Her memory profile was concerning for visual memory dysfunction. The functional MRI of the brain confirmed language activation on the left side for both receptive and expressive speech. An intracarotid amobarbital procedure test demonstrated left-hemispheric language dominance and right-hemispheric dominance for memory (4/10 objects recalled after right injection and 9/10 recalled after left injection).

**Fig. 1:**
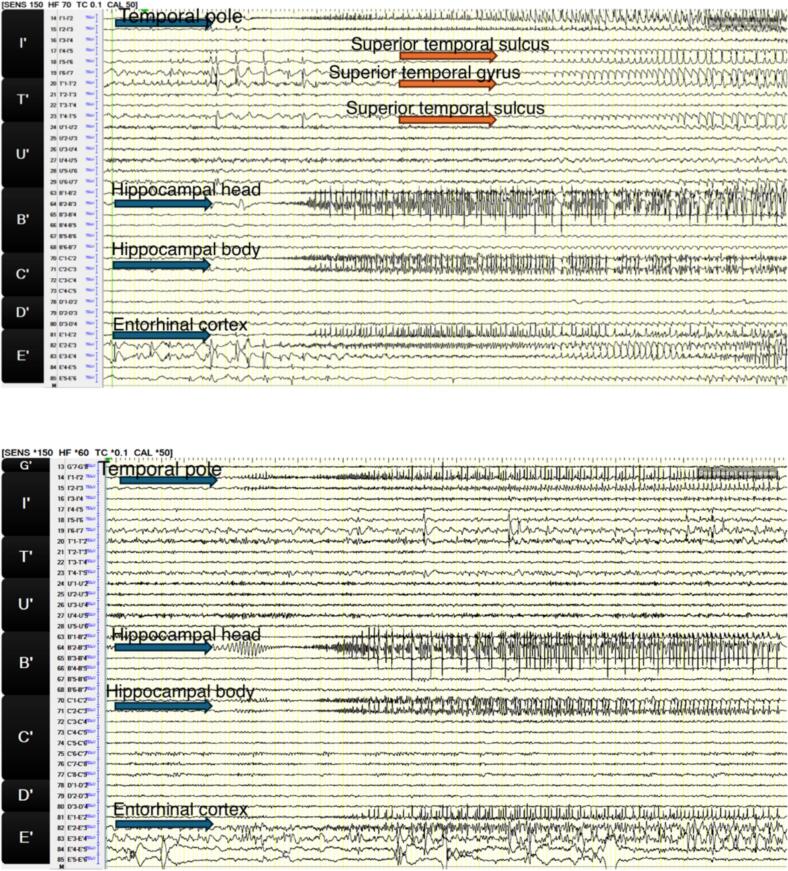
Intracranial EEG [**1a**] demonstrates the typical seizure with RINCH. The onset begins with low-voltage fast activity in the left temporal pole (I’1-2) (**blue arrow**) and rapid propagation to the head (B’1-2) and body of the hippocampus (C’1-3) and the anterior parahippocampal gyrus/entorhinal cortex (E’1-2) (**blue arrows**). Late propagation was observed at 4–5 Hz theta activity to the superior temporal gyrus (I’6-7) and superior temporal sulcus (I’4-5, T’4-5) (**orange arrows**). Intracranial EEG [**1b**] demonstrates the typical seizure without RINCH. The onset with low voltage fast activity onset in the left temporal pole (blue arrow) and rapid propagation to the head and body of the hippocampus and the anterior parahippocampal gyrus (**blue arrows**). There was no propagation to the superior temporal gyrus or the superior temporal sulcus. (For interpretation of the references to colour in this figure legend, the reader is referred to the web version of this article.)

The patient underwent phase 2 SEEG evaluation to identify the EZ and to define the surgical resection limit. Fifteen intracranial depth electrodes were implanted in the left hemisphere, covering the temporal lobe, middle and inferior frontal gyrus, the supramarginal and angular gyrus, anterior and posterior cingulate, insula, and orbitofrontal regions. Scalp EEG electrodes were also placed. Interictal spikes were observed over the temporal pole synchronized with the anterior inferior temporal gyrus, hippocampal head, and body. Thirteen seizures were recorded; of those, 10 were clinical, and 3 were subclinical. All the clinical seizures were stereotyped and were characterized by verbal sounds and hypersalivation, followed by ictal spitting and coughing, 14 s from onset (Video 1). Ictal spitting was observed in all seizures. She then had RINCH movements followed by dystonic posturing in the right upper extremity. RINCH was noted in 6 out of the 10 clinical seizures. Automatisms were noted in the left upper extremity. Postictal cough was also observed. All seizures had low-voltage fast activity onset in the left temporal pole and rapid early propagation to the head and body of the hippocampus on SEEG (Fig. 1 **and** Video 1). Early (<3 s) spread to the anterior parahippocampal gyrus/entorhinal cortex (ictal spitting and coughing) and to the posterior collateral sulcus (cough) was observed. In the next 7 s, the activity spread to the amygdala, and after 10 s, it spread to the superior temporal sulcus (STS) and anterior superior temporal gyrus (STG). Ictal spitting was observed with the spread of the ictal rhythm to the anterior parahippocampal gyrus/entorhinal cortex. Insular contacts (U’1, T’1) were implanted and showed no seizure onset, excluding an insular origin. RINCH was observed once the ictal rhythm spread to the STG and STS, and from there to the inferior frontal gyrus. The patient had 10 clinical seizures, of which 4 seizures did not have RINCH. Comparing seizures with RINCH to seizures without RINCH, seizures without RINCH consistently lacked involvement of STS and STG electrode contacts (Fig. 1). In seizures with RINCH, a time-locked propagation of ictal rhythm to the STS and STG was observed. During cortical stimulation, habitual seizures were provoked by stimulating the hippocampus and the temporal pole. Stimulation of the entorhinal cortex provoked ictal spitting.

SEEG localized the seizure onset zone to the left mesial temporal pole with rapid propagation to the hippocampus and amygdala. The language mapping was negative in the seizure onset zone. The patient underwent left anterior temporal lobectomy (ATL), including the superior temporal gyrus, in the resection.

Neuropathological examination revealed hippocampal sclerosis. She has been entirely seizure-free for one year after the surgery (Engel's class IA and ILAE outcome scale class 1).

## 3 Discussion

In this case, we aim to highlight the electrophysiological correlation of two noteworthy semiological features—ictal spitting and RINCH after detailed invasive EEG evaluation.

RINCH are complex, low-amplitude unilateral, rhythmic, non-clonic movements with milking, grasping, fist clenching, pill-rolling, or larger-amplitude opening and closing motions [7].^7^ These movements can be followed by dystonic posturing of the upper extremity. They have a strong lateralizing value and involve the extremity contralateral to the seizure onset [8].

Researchers have suggested the role of the anterior cingulate and orbitofrontal regions in the pathophysiology of RINCH [10]. Kuba et. al. described 5 patients with RINCH who underwent invasive Video-EEG and had time-locked changes with the activation of either the orbitofrontal cortex or anterior cingulate gyrus [9]. Authors suggested that RINCH may have a similar pathophysiology as ictal dystonia and lateralized ictal immobility of the upper limb, both associated with the widespread epileptic involvement of the contralateral frontal lobe, and are never observed when the ictal activity remains localized to the temporal lobe alone [11].

**Fig. 2:**
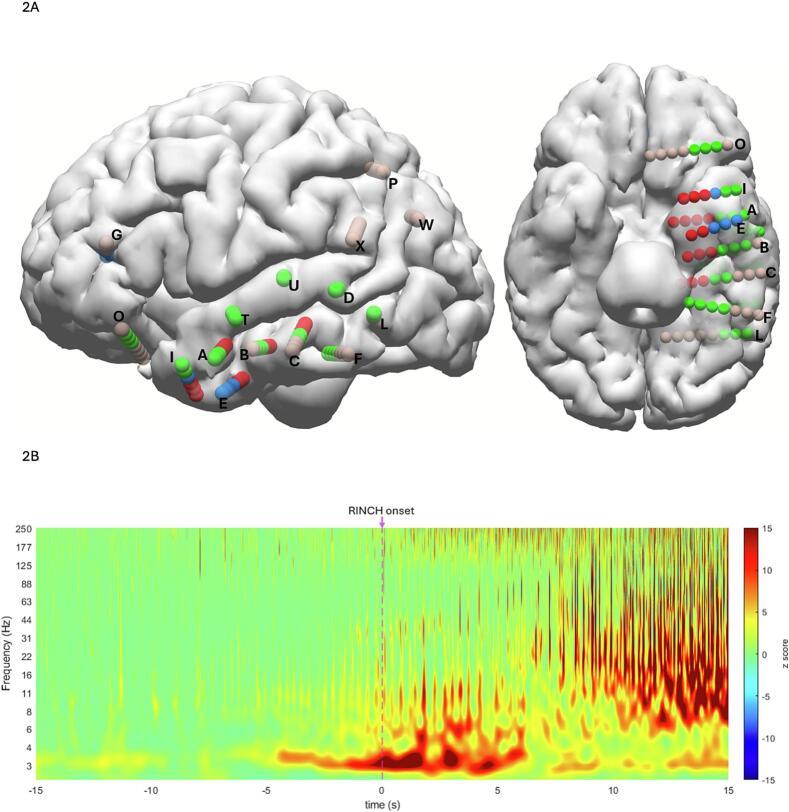
Diagrammatic representation of the seizure onset and propagation patterns for 10 electroclinical seizures classified by semiology (6 with RINCH and 4 without RINCH) [**1a**]. In all 10 seizures, onset and early organization were at the temporal pole and mesial temporal lobe (**red**). All seizures propagated locally (**blue**). Only seizures where RINCH was present propagated more widely in the basolateral temporal lobe (**green**). No seizures propagated to locations labelled light brown within 60 s of seizure onset. This supports the notion that RINCH develops with the recruitment of a broader seizure or functional network.Event-related spectral activity [**1b**]: A time–frequency analysis normalized to pre-ictal baseline activity was generated for each seizure where the behavioral onset of RINCH could be definitively identified. Data were phase locked to RINCH onset (t = 0) and averaged across electro-clinical events (n = 5). Spectral activity in the superior temporal gyrus (electrodes T and U) exhibited oscillatory activity synchronized to RINCH semiology across multiple frequency bands. Other sites of propagation (green locations) were not time-locked to RINCH. (contact T4 pictured, n = 5 events). (For interpretation of the references to colour in this figure legend, the reader is referred to the web version of this article.)

In our patients, it was interesting to note that out of the 10 clinical seizures, 6 were associated with RINCH, and 4 were not. On analysing the propagation pattern in the two groups, it was observed that RINCH always occurred once the ictal rhythm spread to the anterior STG and STS and later propagated to the orbitofrontal regions. The seizures without RINCH did not propagate to those areas. With spectrogram analysis, we demonstrated synchronized STG oscillatory activity time-locked to RINCH onset, supporting its mechanistic role. (Fig. 2). The STG is well connected to motor-executive networks, including the basal ganglia, insula, and prefrontal cortex. Through these networks, the STG integrates auditory and multimodal sensory information and relays this information to the prefrontal and premotor areas, thought to be involved in the generation of semi-automatic motor behaviours. The spread of the ictal rhythm to the STG observed during a seizure may disinhibit motor pathways, particularly those involving hand and arm representations mediated via insula-premotor-basal ganglia loops.

In addition to RINCH, the patient’s seizures were also characterized by hypersalivation, ictal spitting, ictal cough, throat clearing, postictal cough, and postictal nose wiping. Ictal hypersalivation can be observed in lesions in the fronto-orbital cortex and cingulate gyrus, insula, operculum, Rolandic area, and mesial temporal structures [12,13]. However, no clear lateralization has been reported, though most reports mention non-dominant lobe involvement. Ictal spitting, as seen in our patient, has been described in non-dominant TLE [4,[13], [14], [15], [16], [17]]. In our case, insular contacts were not involved in the propagation. Ictal spitting was consistently time-locked to entorhinal/parahippocampal spread. No ictal onset was recorded in the insula, excluding it as the generator. Rare reports link ictal spitting in dominant TLE to entorhinal cortex activation, as observed in our case [[18], [19], [20]]. Ictal cough and throat clearing may be seen in temporal and extratemporal seizures, but have a temporal lobe origin if they are a regular semiological event, as observed in our case [5]. Post-ictal nose wiping, linked to insular and amygdala involvement, is not specific to temporal lobe seizures only and may reflect seizure propagation [6]. Nonetheless, it has been shown to be ipsilateral to seizure onset in 86.5 % of TLE patients [14]. PIVS often suggests a possible non-dominant temporal lobe involvement. However, our case highlights the observation that PIVS can be a feature of TLE but does not have any lateralizing importance.

The histopathological diagnosis of hippocampal sclerosis and the post-operative seizure freedom establish the accurate prediction of the EZ in this case. As per the recent grading system proposed after an expert consensus for assessing the confidence in the epileptogenic zone, a class IA and class I postoperative seizure outcome is associated with a very high confidence level in the localization of the reported EZ [10]. Thus, the successful post-surgical outcome in our case strengthens the anatomic-electro-clinical correlations of the PIVS and RINCH, which were delineated through invasive stereo-EEG. Though seizure freedom after ATL supports correlation, it does not, however, prove causality or the generator of the semiology.

## 4 Conclusion

This case provides rare SEEG evidence linking *peri*-ictal vegetative and motor automatisms to distinct cortical regions, with ictal spitting arising from entorhinal cortex engagement and RINCH associated with superior temporal gyrus activity. The results confirm RINCH as a lateralizing sign, in contrast to PIVS, which lacks such significance.

## CRediT authorship contribution statement

**Divya Nagabushana:** Writing – review & editing, Writing – original draft, Formal analysis, Data curation, Conceptualization. **Francesco Pucci:** Writing – review & editing, Supervision, Formal analysis, Data curation. **Huan Huynh:** Writing – review & editing, Validation, Supervision, Formal analysis. **Julia Bodnya:** Writing – review & editing. **Anna Serafini:** Writing – review & editing, Writing – original draft, Supervision, Formal analysis, Data curation, Conceptualization.

## Declaration of competing interest

The authors declare that they have no known competing financial interests or personal relationships that could have appeared to influence the work reported in this paper.
