# Supplementary material for: Electro-clinical correlation of rinch and peri-ictal vegetative symptoms
Source: Epilepsy Behav Rep. 2025 Oct 9;32:100831. doi: 10.1016/j.ebr.2025.100831 (PMC12547204; doi:10.1016/j.ebr.2025.100831)
Supplement: Supplementary Data 1 [file mmc1.docx]

**Electro-Clinical Correlation of RINCH and Peri-Ictal Vegetative Symptoms**

Divya Nagabushana^1^, Francesco Pucci^2^, Huan Hyunh^1^, Julia Bodnya^1^, Anna Serafini^1^

**^1^** Epilepsy Division, Department of Neurology and Rehabilitation, University of Illinois Chicago, Chicago, USA

**^2^** Department of Neurosurgery, University of Illinois Chicago, Chicago, USA

**Corresponding author:** Anna Serafini, M.D

Epilepsy section chief

Director Epilepsy Monitoring Unit

Associate Professor of Neurology – Epilepsy

Department of Neurology and Rehabilitation
University of Illinois Chicago
912 S. Wood St,
Chicago, IL USA 60612

Tel: [(312) 413-8114](tel:(312)%20413-8114)
Fax: [(312) 996-4169](tel:(312)%20996-4169)

Email: [serafini@uic.edu](mailto:serafini@uic.edu)

**Running title:** RINCH and peri-ictal vegetative symptoms in TLE

**Keywords:** ictal spitting; rhythmic ictal non-clonic hand motions; temporal lobe epilepsy; peri-ictal vegetative symptoms; invasive EEG; automatisms

**Video keywords:** Focal non-idiopathic temporal; hippocampal sclerosis; RINCH automatisms; ictal spitting; temporal mesiolateral

**Number of pages:** 9

**Word count of text:** 1583

**Word count of abstract:** 223

**No. of references:** 20

**No. of figures:** 2

**No. of video sequences:** 1

**No. of tables:** nil

**Author Contributions**: DN, AS, FP, JB, and HH contributed to the conception of the work, acquisition, analysis, interpretation of data, and preparation of the initial draft of the work. AS, HH, FP and JB revised it critically, and the final version was approved by all.

**Declaration of patient consent**

The authors certify that they have obtained all appropriate patient consent forms. In the form, the patient(s) has/have given his/her/their consent for his/her/their images and other clinical information to be reported in the journal.

**Data availability:** Data is available with the authors. Anonymized data will be shared if ethically indicated.

**Source of support / funding:** nil

**Financial support and sponsorship**

Nil.

**Conflicts of interest**

There are no conflicts of interest.
